# Understanding of Young People About COVID-19 During Early Outbreak in Indonesia

**DOI:** 10.1177/1010539520940933

**Published:** 2020-07-09

**Authors:** Devina Adella Halim, Andree Kurniawan, Fransisca Handy Agung, Stella Angelina, Claudia Jodhinata, Sharleen Winata, Frenstan, Felix Wijovi, Cindy Monika Agatha

**Affiliations:** 1 Faculty of Medicine, Pelita Harapan University, Tangerang, Banten, Indonesia; 2 Internal Medicine Department, Faculty of Medicine, Pelita Harapan University, Tangerang, Banten, Indonesia; 3 Child and Adolescent Health Department, Faculty of Medicine, Pelita Harapan University, Tangerang, Banten, Indonesia

**Keywords:** COVID-19, coronavirus, outbreak, young people, understanding, knowledge, Indonesia

## Abstract

To control the spread of COVID-19 transmission, Indonesia government has broadcasted information about the pandemic. The aim of this study is to evaluate the understanding of young people, about COVID-19 during the early outbreak in Indonesia. An online-based cross-sectional data collection was conducted from adolescents aged 10 to 25 years, based on 10 questions regarding general COVID-19 information. There were 355 subjects from 25 out of 34 Indonesian provinces who participated in the study, with mean age of 19.93 ± 2.91 years. Better understanding was found in the female and higher-income population. Largely, the subjects got the information from social media, instead of the official government site for COVID-19. Lack of understanding about crucial preventive measures was found, such as handwashing and physical distancing. In conclusion, the participants have an overall moderate-good initial understanding toward COVID-19 during the early outbreak in Indonesia. These results can be used as baseline data for development of awareness measurement tools.

## Introduction

Coronavirus disease 2019 (COVID-19) has become a global pandemic including all provinces of Indonesia. Information on the pandemic have been made available by the government as it was important to improve everyone’s awareness and understanding, especially in young people.^1^ Indonesia’s population is young with 25% younger than 15 years and 49% younger than under 25 years of age.^2^ There has been no studies of the understanding and awareness of young people regarding COVID-19.

## Methods

A cross-sectional study design was conducted during April 22 to 25, 2020. We relied on social media to distribute a poster containing our study’s details. The eligible participants were Indonesian residents aged 10 to 25 years, and those who were studying or active in health science fields were excluded. The participants were required to complete an e-consent form and then a self-reported questionnaire. The questionnaire was generated based on information provided by the government and reviewed by experts in field, followed by a field trial with 40 participants aged 18 to 22 years. The open-ended questions were given to evaluate respondents’ knowledge of virology, clinical presentation, risk factors, prevention, control, symptoms handling, and one yes-no question regarding their awareness of the official government website for COVID-19 information. Each correct answer was valued 10 points. Those who scored 60 or above were considered as having good knowledge. Top five answers from each question are described.

## Results

A total of 355 participants, from 25 out of the 34 Indonesian provinces, completed the questionnaire. Most participants resided in the western and central Indonesia, and only six participants were from the eastern provinces. The average age of the participants was 19.93 ± 2.9, most were male (81.1%), held bachelor’s degree or above (44.2%), and students (52.39%).

In the first knowledge question, the answers given for the cause of the infection included animals, food, unclean environment, bacteria, and few did not know. When asked about method of spread, the answers included through droplets, direct contact, touching contaminated items, touching eyes, nose, and mouth. The majority of the respondents answered correctly for COVID-19 symptoms and population at risk. Behaviors to prevent the spread of COVID-19 were known only by a minority of the population; physical distancing was only known by around half of the study subjects, and even less for its detailed definition, and only one third of the population mentioned handwashing. Confirming symptoms that were mentioned included temperature checks, physical examination, and going to health care providers. Answers like “swab test,” “rapid test,” and “PCR” were included in virology. More than one half were aware of the existence of government website. More details can be seen in Table 1.

**Table 1.** Basic Knowledge of COVID-19.

Answers	N	%
1. What is causing COVID-19?		
Virus	200	56.3
Non-virus	180	50.7
2. How can COVID-19 be transmitted?		
In accordance to the fact	215	60.6
Not in accordance to the fact	199	56.1
3. What are the symptoms of COVID-19?		
Fever	298	83.9
Cough	272	76.6
Shortness of breath	180	50.7
Runny nose	141	39.7
Sore throat	81	22.8
Others	134	37.7
4. Who is at risk of experiencing serious illness due to COVID-19?		
Older adults with comorbidities	202	56.90
People with weakened immune systems	51	14.37
Health care providers	26	7.32
Pregnant women	1	0.28
Others	214	60.28
5. How to prevent the spread of COVID-19?		
Physical distancing	179	50.4
Washing hands	113	31.8
Wearing personal protective equipment	72	20.3
Applying healthy lifestyle	52	14.6
Avoid touching on face	12	3.4
6. What is social (physical) distancing?		
Increasing the physical space between people	155	43.7
I don’t know	51	14.4
Avoiding crowds	45	12.7
Reducing contact and/or interaction with others	35	9.9
Staying at home	34	9.6
7. If you have fever and cough, where will you go to look for treatment?		
Primary health care facilities	230	64.79
Hospital	132	37.18
Call COVID-19 hotline	3	0.84
Self-medication	31	8.73
Not seeking any medical care	13	3.66
8. What should you do when you’ve come in contact with suspect or confirmed case of COVID-19 patient?		
Practice hygiene	143	40.3
Go to health care facilities	106	29.9
Self-isolation	50	14.1
Wear mask	19	5.3
9. What examination should be conducted once you experience symptoms suggestive of COVID-19?		
Laboratory tests	81	22.8
Confirming symptoms	80	22.5
I don’t know	62	17.5
Virology	43	12.1
Radiology	19	5.3
10. Are you aware that there is a government’s official website for COVID-19?		
Yes	199	56.1
No	156	43.9

The most used source for retrieving COVID-19 information was social media (33.5%); followed by television (33.2%); news websites (29.0%); family, friends, or relatives (11.3%); and medical staff (2.2%). The overall understanding among urban and rural populations was inadequate and was higher in Java compared with other provinces and was associated with higher incomes and being female.

## Discussion

Our data demonstrated that Indonesian young people had some understanding of the ongoing outbreak, and many of the answers were in accordance with the information provided by the government. Physical distancing and handwashing practice appeared to be poorly understood. In contrast, study in China revealed that fewer than 5% Chinese residents went to crowded places during the pandemic.^3^ While based on the national survey in 2013, less than half of the residents (47%) had proper handwashing behavior.^4^ A study to look further on how we can improve handwashing practice is recommended.

Although more than half of the subjects were aware of the government’s official website for COVID-19, very few respondents sought information from it. Instead, these young people preferred to gain information through social media. Internet-based data can play a major role in real-time reporting to empower active public health surveillance systems.^5^ Public awareness campaigns regarding COVID-19 and the need for early health care visits when presenting with symptoms have been successful but have yet to expand for the appropriate steps after they had contact with suspected or confirmed COVID-19 patients.

The limitation of the study was that it was conducted online; thus, rural people or those living without internet access might not have participated. Future studies will require a more robust sampling method that is representative of all regions.

## Conclusion

All young people of Indonesia are aware of the COVID-19 pandemic, but knowledge about preventive measures is inadequate. Further research is needed on communicating messages on preventive measures, such as physical distancing and handwashing.
